# Prescription of suboptimal statin treatment regimens: a retrospective cohort study of trends and variation in English primary care

**DOI:** 10.3399/bjgp20X710873

**Published:** 2020-06-30

**Authors:** Helen J Curtis, Alex J Walker, Brian MacKenna, Richard Croker, Ben Goldacre

**Affiliations:** The DataLab, Nuffield Department of Primary Care Health Sciences, University of Oxford, Oxford.; The DataLab, Nuffield Department of Primary Care Health Sciences, University of Oxford, Oxford.; The DataLab, Nuffield Department of Primary Care Health Sciences, University of Oxford, Oxford.; The DataLab, Nuffield Department of Primary Care Health Sciences, University of Oxford, Oxford.; The DataLab, Nuffield Department of Primary Care Health Sciences, University of Oxford, Oxford.

**Keywords:** cardiovascular diseases, lipids, primary health care, retrospective studies, statins

## Abstract

**Background:**

Since 2014 English national guidance recommends ‘high-intensity’ statins, reducing low-density lipoprotein (LDL) cholesterol by ≥40%.

**Aim:**

To describe trends and variation in low-/medium-intensity statin prescribing and assess the feasibility of rapid prescribing behaviour change.

**Design and setting:**

A retrospective cohort study using OpenPrescribing data from all 8142 standard NHS general practices in England from August 2010 to March 2019.

**Method:**

Statins were categorised as high- or low-/medium-intensity using two different thresholds, and the proportion prescribed below these thresholds was calculated. The authors plotted trends and geographical variation, carried out mixed-effects logistic regression to identify practice characteristics associated with breaching of guidance, and used indicator saturation to identify sudden prescribing changes.

**Results:**

The proportion of statins prescribed below the recommended 40% LDL-lowering threshold has decreased gradually from 80% in 2011/2012 to 45% in 2019; the proportion below a pragmatic 37% threshold decreased from 30% to 18% in 2019. Guidance from 2014 had minimal impact on trends. Wide variation was found between practices (interdecile ranges 20% to 85% and 10% to 30% respectively in 2018). Regression identified no strong associations with breaching of guidance. Indicator saturation identified several practices exhibiting sudden changes towards greater guideline compliance.

**Conclusion:**

Breaches of guidance on choice of statin remain common, with substantial variation between practices. Some have implemented rapid change, indicating the feasibility of rapid prescribing behaviour change. This article discusses the potential for a national strategic approach, using data and evidence to optimise care, including targeted education alongside audit and feedback to outliers through services such as OpenPrescribing.

## INTRODUCTION

Statins are very widely used to control serum cholesterol and reduce the risk of cardiovascular disease (CVD), with up to 7 million of the UK population (64.6 million) taking them in 2014.^1^ This makes statins the most commonly prescribed class of drugs in England, with 72.5 million prescriptions costing >200 million GBP dispensed during 2017.^2^^,^^3^ The 2014 guidance on lipid modification by the National Institute for Health and Care Excellence (NICE)^4^ recommends the use of high-intensity statins, capable of reducing low-density lipoprotein (LDL) cholesterol by ≥40%, for both primary and secondary prevention.^5^ This recommendation was made on the basis that higher-intensity treatment offers substantially greater reduction in cardiovascular risk, with similar adverse effects and cost. All patients with >10% 10-year risk of CVD are to be offered statins under NICE guidance.^4^ If someone were to have a 15% 10-year risk, their risk would reduce to 9% with a recommended high-intensity statin.^6^ The high-intensity treatment options available in the UK are atorvastatin ≥20 mg; simvastatin 80 mg; and rosuvastatin ≥10 mg. Fluvastatin is medium-intensity at its highest dose; and pravastatin is low-intensity at all doses.

Two retrospective analysis studies carried out in UK patient-level datasets indicate that a huge shift in treatment would be required to meet the new recommendations: in 2013, of patients eligible for secondary prevention, only 24% were receiving high-intensity statins;^7^ and, in 2014, 31% of secondary prevention patients received high-intensity statins, with 21% not receiving statins at all.^8^ This second study also noted that only 6% of these patients were receiving statin therapy fully in line with the new guidelines for secondary prevention (atorvastatin 80 mg or equivalent); similarly, for patients eligible for primary prevention, only 15% were on high-intensity statins (minimum atorvastatin 20 mg or equivalent).

The authors’ group runs OpenPrescribing. net, an online service that gives free and open access to monthly prescription data and charts describing various treatment choices at every general practice in England, with over 130 000 unique users during the past year. This service includes a standard ‘audit and feedback’ measure that describes the prescribing of low-/medium-intensity statins, as a proportion of all statin prescribing, at each practice (https://www.openprescribing.net/measure/statinintensity). The authors were concerned to find that compliance with NICE guidance on statin prescribing was extremely varied. Therefore, they set out to describe trends and variation in the proportion of all statin prescribing in English primary care that breaches this guidance; to identify factors associated with breaching; and to assess the feasibility of prescribing change by ascertaining whether there were individual practices that had rapidly implemented substantial changes.

**Table:** How this fits in

English national guidance recommends the use of high-intensity statins, capable of reducing low-density lipoprotein (LDL) cholesterol by ≥40%. Studies in subsets of general practice data have shown that compliance at the time of guideline release was low, but has not been documented since. The present study of the complete population of English general practice shows improving guideline compliance, but that prescribing of low-intensity statins remains common, with 45% of prescriptions below the recommended strength, and there is very substantial variation between practices. Some practices have exhibited rapid positive change in prescribing, which indicates that better guideline compliance could readily be achieved. The authors have produced a live-data tool allowing anyone to explore any practice’s current statin prescribing behaviour.

## METHOD

### Study design

This was a retrospective cohort study of statin prescribing behaviour using routinely collected primary care prescribing data. Outcomes were not pre-specified.

### Setting

NHS primary care in England was the setting, including all general practices (*N* = 8142) with statin prescriptions dispensed from August 2010 to March 2019.

### Data sources

Data from OpenPrescribing, which imports national prescribing data published by NHS Business Services Authority (BSA), were used along with other datasets for practice characteristics, as previously described.^9^^,^^10^ Prescribing data record the number of times each individual drug presentation was prescribed in all primary care settings in England (and dispensed in the community), every month since August 2010.^11^ 'Items' corresponds to number of prescriptions dispensed, whereas 'quantity' is the total number of tablets or millilitres, for example. Statin treatment is typically a single tablet taken once a day, meaning that tablet strengths are an appropriate surrogate for statin dose.

### Data processing

Monthly data were extracted from August 2010 to March 2019 inclusive for all statins (see Supplementary Table S1). Data extraction was restricted to general practices (setting code ‘4’), excluding atypical settings, for example, prisons and out-of-hours services.^12^ The number of patients registered per practice from NHS Digital^13^ were obtained. Statins were classified by strength, with rosuvastatin <10 mg, atorvastatin <20 mg, and simvastatin <80 mg classed as low-/medium-intensity according to NICE guidelines; and a more pragmatic classification based on a statin intensity threshold of 37% reduction in LDL, with rosuvastatin 5 mg, atorvastatin 10 mg, and simvastatin 40 mg additionally grouped with high-intensity formulations (see Supplementary Table S1 for BNF codes).

### National trends

Monthly total statin items, the proportion of low-/medium-intensity statins, and the rate of low-/medium-intensity statin items prescribed per 1000 registered patients were calculated and time trend charts plotted.

### CCG-level variation

Each practice in England is a member of a regional clinical commissioning group (CCG), which oversees and funds their medication prescribing. The proportion of low-/medium-intensity statins for each CCG in 2018 was calculated and displayed on a map.

### Practice-level variation

The statin prescribing rate per 1000 registered patients and the proportion of low-/medium-intensity in each practice were calculated. Deciles and centiles on time trends charts are displayed. This was repeated for the proportion of all statin tablets (quantity) prescribed as each common formulation of atorvastatin and simvastatin, for example, 20 mg and 40 mg (liquid presentations were excluded).

### Logistic regression

A mixed-effects logistic regression model was created to assess the practice factors associated with low-/medium-intensity statin prescribing in 2018. The fixed-effect variables, selected a priori from clinical interest and data availability, were: proportion of patients registered aged >65 years; proportion of patients with a self-reported long-term health condition; practice list size (NHS Digital); Index of Multiple Deprivation score (each sourced from Public Health England);^14^ Quality and Outcomes Framework (QOF) score;^15^ and rural/urban location of practice postcode.^16^ CCG was included as a random effect. Practices with missing data were dropped from that part of the analysis. Continuous variables were categorised into quintiles to allow for nonlinearity of effects and to improve intelligibility of results. The main outcome was low-/medium-intensity statin (<40% LDL reduction) prescriptions as a proportion of all statin prescriptions, transformed using a conditional logit transformation.^17^ This can be conceived of as a logistic regression analysis where each prescription is a binary choice to give either guideline-compliant or non-compliant treatment. Odds ratios (OR) and 95% confidence intervals (CI) for each of the fixed-effect variables were calculated, as well as an R-squared value (along with the significance level) to describe the degree of variance associated with CCG membership.

### Practices that have changed quickly

The proportion of low-/medium-intensity (<40% LDL reduction) statin prescribing, for each month, for each practice was calculated. The authors applied their previously described indicator saturation method,^18^ to detect the timing, slope, and magnitude of changes in prescribing. This is an automated, hypothesis-blind method of detecting sudden changes in time series data. Example practices with large and rapid changes were identified and the time series plotted.

### Software and reproducibility

Data management was performed using Python 3 and Google BigQuery, with analysis carried out using Stata (version 14.2) and/or Python 3. All data were shared openly online alongside all code for data management and analysis: https://github.com/ebmdatalab/statins-dose-paper.

## RESULTS

### Study population

All 8142 standard general practices in England were included across the entire time period. In 2018 there were 7210 practices, organised into 195 local CCGs.

### National trends

**Figure 1.**
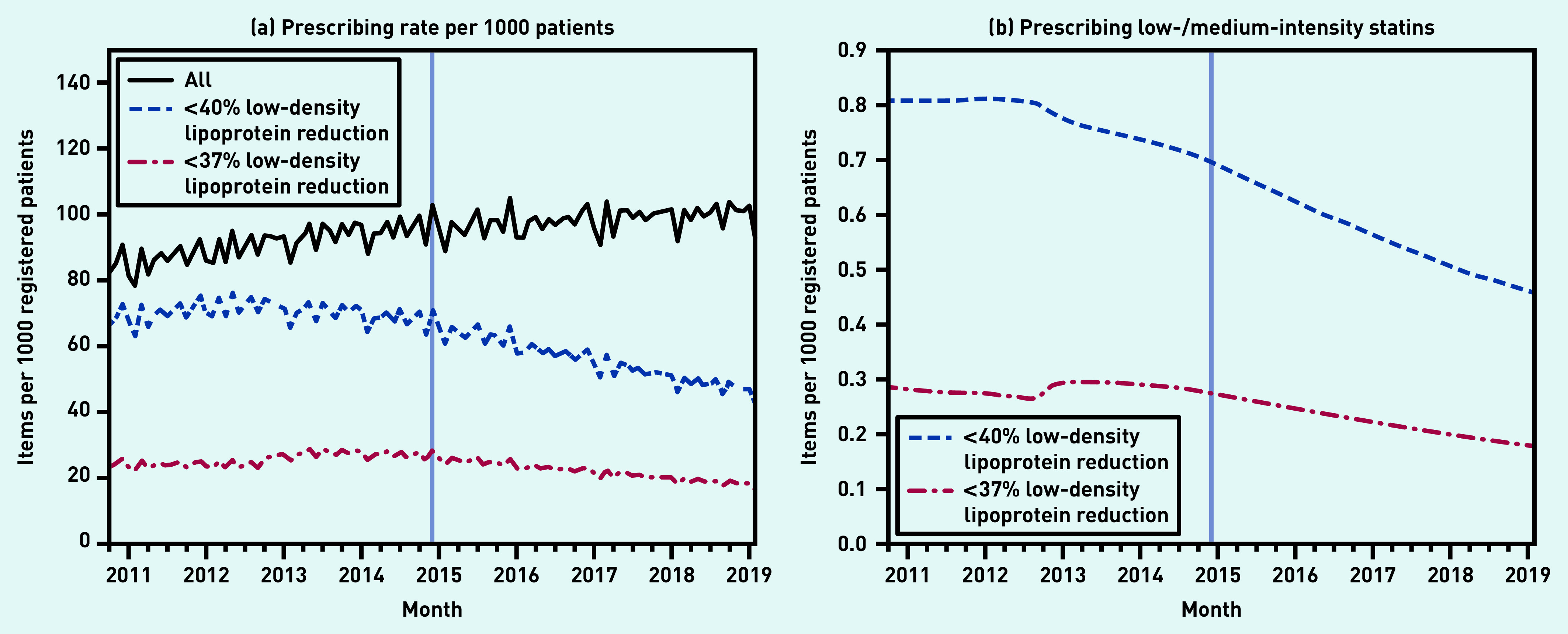
**Monthly statin prescribing across English NHS practices: a) total statin items prescribed per 1000 registered patients, and those of low-/medium-intensity, at both <40% and <37% intensity thresholds; b) proportion of statin items prescribed that were of low-/medium-intensity, including both intensity thresholds. Vertical line indicates release of National Institute for Health and Care Excellence guidance, July 2014.**

Overall, statin prescribing increased from around 85 to 90 items per 1000 patients per month in 2011/2012 to around 100 items in 2018/2019 (Figure 1a). Low-/medium-intensity statins, according to NICE criteria (≥40% reduction in LDL cholesterol), made up 80% of statin prescriptions in 2011/2012, declining to approaching 45% in 2019, at 5.4 percentage points per year (Figure 1b). When measuring the proportion under a pragmatic 37% reduction threshold, to account for patients not being reviewed/switched if they were already on statins very close to the NICE threshold, the proportion declined from a peak of 30% in 2013 to 18% in 2019 (Figure 1b). Notably, prescribing of atorvastatin 10 mg, 20 mg, and 40 mg, and simvastatin 20 mg underwent a sharp increase in 2012, coinciding with a rapid reduction in simvastatin 40 mg (see Supplementary Figure S1). Thereafter, prescribing of high-intensity atorvastatin (20–80 mg) increased, whereas atorvastatin 10 mg levelled off and all simvastatin declined.

### CCG-level variation

**Figure 2.**
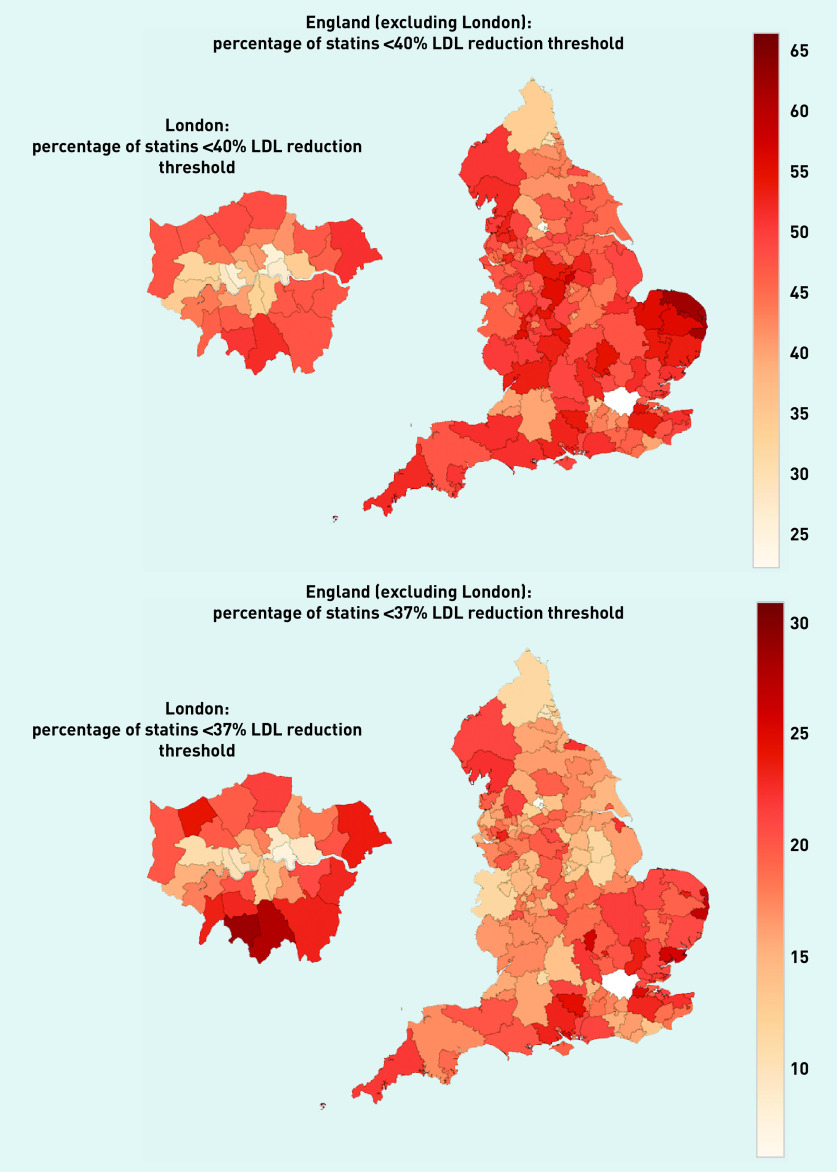
**Percentage of all statin items prescribed in low-/medium-intensity formulations(<40% LDL reduction and <37% LDL reduction) across each clinical commissioning group in England, 2018. LDL = low-density lipoprotein.**

Among England’s CCGs, the proportion of statins prescribed in low-/medium-intensity formulations in 2018 ranged widely, approximately 25–65%, or 7–31% under the 37% LDL reduction threshold, with the closest compliance with guidelines in Central London and around Bradford (Figure 2).

### Practice-level variation in statin prescribing

**Figure 3.**
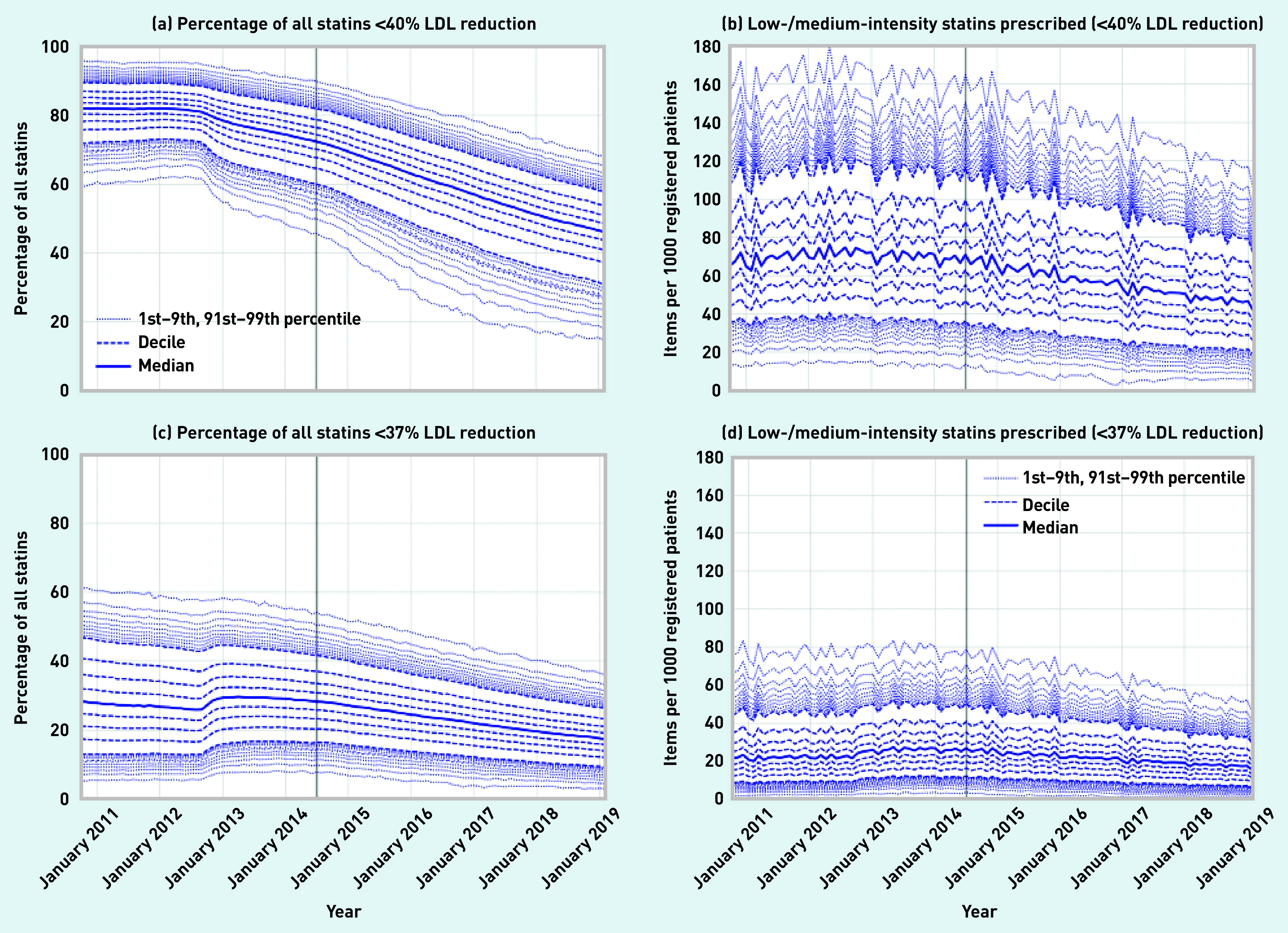
**Monthly prescribing rates of low-/medium-intensity statin across England’s practices from 2011 to 2019. (a, c) Percentage of all statin items under given intensity threshold. (b, d) Number of items under given intensity threshold prescribed per 1000 registered patients. Vertical line indicates release of National Institute for Health and Care Excellence guidance, July 2014.**

The national decline in low-/medium-intensity statins was reflected in individual practices across all deciles, but variation increased slightly over time (Figure 3a). Nonetheless, in 2018, 10% of practices still prescribed >60% of statins as low-/medium-intensity (interdecile range 20% to 85%; Figure 3a). The decline in absolute prescribing rate per 1000 population was less pronounced (Figure 3b), and with a very wide variety in performance: in 2018, 10% of practices prescribed ≤25 per 1000 patients per month; whereas the top 10% prescribed ≥80. For comparison, the monthly prescribing rate for all statins' interdecile range was 50 to 160 (see Supplementary Figure S2a). For statins below the 37% threshold, variation narrowed, with interdecile range reducing from almost 30% to around 20% (interdecile range in 2018 between 10% and 30%; Figure 3c).

### Practices that have changed quickly

**Figure 4.**
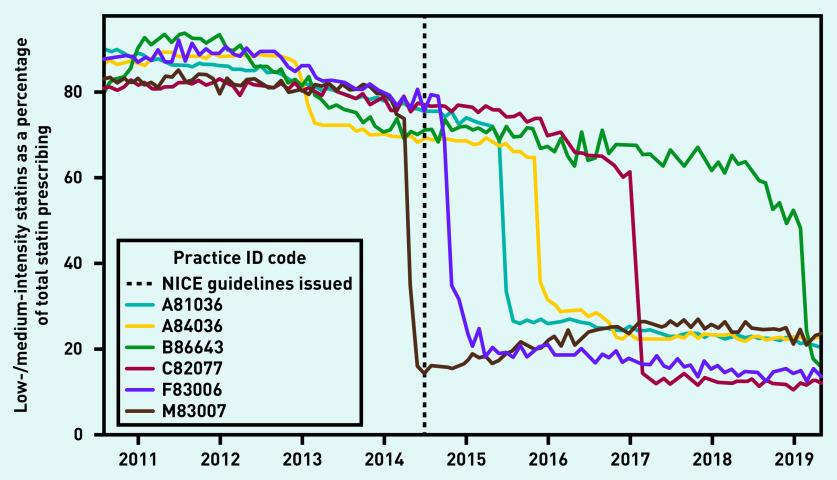
***Examples of practices that have rapidly reduced their percentage of low- and medium-intensity statin (<40% LDL reduction) prescribing over the latest 5 years. Vertical line indicates release of NICE guidance, July 2014.*** ***LDL = low-density lipoprotein. NICE = National Institute for Health and Care Excellence.***

The indicator saturation method found a substantial number of practices demonstrating very rapid changes in statin prescribing towards greater guideline compliance. For example, since 2014 there were 96 practices (from 57 CCGs) with a change of >5 percentage points per month (compared with the national rate of 5.4 per year), amounting to a total change of >25 percentage points. Examples of practices with the quickest changes are illustrated in Figure 4.

### Regression

Practice factors associated with the proportion of low-/medium-intensity statin prescribing in 2018 were modelled and shown in Table 1. Multivariable regression indicated that the only variable with any meaningful association was patient age: practices with the highest proportion of patients aged >65 years were slightly more likely to prescribe a greater proportion of low-/medium-intensity statins (multivariable OR for youngest versus oldest: 1.22, 95% CI = 1.17 to 1.28). All other factors had ORs close to 1 (range 0.84 to 1.03). However, the CCG to which a practice belonged (as a random effect) was significantly associated with prescribing (*P*<0.001) and accounted for 25.7% of variation. A total of 350 practices were excluded from the multivariable analysis owing to missing data.

**Table 1.** Unadjusted and adjusted estimates for practice-level prescribing of low-/medium-intensity statins as a proportion of all statins from logistic regression analysis

**Practice factors**	**Median proportion of low-/medium-intensity statin prescribing**	**Univariable logistic regression odds ratio (95%CI)**		**Multivariable logistic regression odds ratio (95%CI)**	
**Patients aged** >**65 years, %^a^**					
0.0–10.8	0.16	Ref		Ref	
10.8–15.4	0.18	1.22	(1.18 to 1.26)	1.13	(1.09 to 1.16)
15.4–18.9	0.19	1.28	(1.24 to 1.32)	1.16	(1.12 to 1.20)
18.9–22.7	0.20	1.31	(1.27 to 1.35)	1.18	(1.14 to 1.23)
22.7–89.8	0.20	1.31	(1.27 to 1.36)	1.22	(1.17 to 1.28)

**Patients with a long-term health condition, %^a^**					
10.0–43.8	0.17	Ref		Ref	
43.8–49.4	0.19	1.13	(1.09 to 1.16)	1.02	(0.99 to 1.05)
49.4–53.6	0.19	1.16	(1.12 to 1.20)	1.03	(1.00 to 1.06)
53.6–58.3	0.19	1.13	(1.09 to 1.17)	1.01	(0.97 to 1.04)
58.3–92.5	0.18	1.11	(1.07 to 1.14)	1.00	(0.97 to 1.04)

**Practice list size, 1000s^a^**					
0.0–4.1	0.18	Ref		Ref	
4.1–6.1	0.18	1.00	(0.96 to 1.03)	1.01	(0.98 to 1.04)
6.1–8.6	0.18	1.03	(0.99 to 1.06)	1.01	(0.98 to 1.04)
8.6–11.8	0.19	1.01	(0.98 to 1.05)	0.98	(0.95 to 1.01)
11.8–72.5	0.19	1.06	(1.03 to 1.10)	0.99	(0.96 to 1.02)

**Urban/rural setting**					
Urban, major conurbation	0.18	Ref		Ref	
Urban, minor conurbation	0.17	0.99	(0.93 to 1.05)	0.99	(0.88 to 1.11)
Urban city and town	0.20	1.15	(1.13 to 1.18)	1.02	(0.97 to 1.07)
Rural town and fringe	0.20	1.15	(1.11 to 1.19)	0.96	(0.91 to 1.02)
Rural village and dispersed	0.17	1.00	(0.95 to 1.06)	0.84	(0.79 to 0.90)

**IMD quintile**					
5 (least deprived)	0.20	Ref		Ref	
4	0.20	0.96	(0.93 to 1.00)	0.99	(0.96 to1.02)
3	0.19	0.92	(0.89 to 0.95)	0.98	(0.95 to 1.01)
2	0.18	0.86	(0.84 to 0.89)	0.96	(0.93 to 1.00)
1 (most deprived)	0.16	0.76	(0.74 to 0.79)	0.89	(0.85 to 0.93)

**QOF score^a^**					
14–523	0.19	Ref		Ref	
523–541	0.19	1.00	(0.97 to 1.03)	0.99	(0.96 to 1.01)
541–550	0.19	0.99	(0.96 to 1.03)	0.98	(0.95 to 1.01)
550–557	0.18	0.99	(0.96 to 1.02)	0.97	(0.94 to 1.00)
557–559	0.19	1.01	(0.97 to 1.04)	0.96	(0.93 to 0.99)

a Figures are rounded. IMD = Index of Multiple Deprivation, QOF = Quality and Outcomes Framework.

## DISCUSSION

### Summary

NICE guidance recommends high-intensity statins. Compliance is improving but prescriptions of lower-intensity statins remain extremely common. Using NICE criteria (40% reduction in LDL cholesterol), low-/medium-intensity statins fell from 80% of statins prescribed in 2011/2012 to 45% in 2019, at a rate of 5.4 percentage points per year. Against more permissive criteria (<37% LDL reduction), the proportion fell from 30% in 2013 to 18% in 2019. Release of the NICE guidance in 2014 had minimal impact on these trends. Substantial variation in prescribing behaviour exists between practices, with an interdecile range 20% to 85% for NICE criteria, and 10% to 30% for the permissive criteria. Examples of practices rapidly changing towards greater guideline compliance were found in this study, demonstrating that this is potentially achievable more widely.

### Strengths and limitations

The inclusion of almost the entire population of England minimised the potential for bias in this study. Data sourced from pharmacy claims were highly accurate and included all dispensed medication. Both the NICE cut-off point of 40% LDL reduction, and a more permissive cut-off of 37%, were examined to account for the very widespread use of statins falling just under the NICE efficacy threshold before release of these guidelines. Pragmatically, when reviewing patients, GPs likely prioritise those who most substantially breach the NICE guidance.

It was not possible to stratify by risk or comorbidities in the data available, but the present work represents a pragmatic analysis for every practice in the country, which can allow areas to be prioritised and further investigated locally. The proportion of statins breaching guidance were used, rather than the absolute numbers, to account for variation in the number of people per practice receiving statins. In some cases lower-intensity statins will be prescribed appropriately, as per the exceptions in the guidance, for example, with intolerance or perceived intolerance of higher doses.^4^ However, prevalence of intolerance is likely around 10% to 11% at most,^19^^,^^20^ and certainly much less than the proportion of low-/medium-intensity statins prescribed (45%). Tolerance rates are only slightly lower in high-intensity compared with low-intensity statins.^21^^,^^22^ Furthermore, variation in prevalence of intolerance or other factors are unlikely to match the scale of variation in prescribing between practices, especially given the very high numbers involved. This could be assessed by interrogating richer electronic health record (EHR) data; however, concerns regarding statin intolerance are unlikely to be recorded consistently as structured data.

### Comparison with existing literature

The present findings are consistent with previous work on smaller populations in UK patient-level datasets. For example, before guideline release, 24–31% of largely secondary prevention patients and 15% of primary prevention patients received high-intensity statins;^7^^,^^8^ and, among patients initiating statins for secondary prevention 2010–2013, 74% were started on ‘moderate’ intensity (27–43% reduction, including atorvastatin 20 mg) and 23% on high-intensity (>42% reduction).^23^ Consistent with this, the present data showed approximately 70–75% of statins were low-/medium-intensity in 2014. The present study also revealed that the decline in use of low-/medium-intensity statins began before the 2014 guidelines.

As statins are taken long term, newly initiated prescriptions represent a minority of the total. The association found with patient age is consistent with some patients continuing to take statins initiated pre-2014 without review; and a greater number of older patients represents a higher workload involved in undertaking reviews. There may also be some avoidance of greater perceived risk of adverse effects in older patients.

Low levels of high-intensity statin usage have also been reported across Europe and worldwide, with a substantial proportion of patients not achieving target cholesterol levels.^24^^,^^25^ In the US, adherence to similar guidance on statins was strongly associated with geography, indicating that local policy or culture plays an important role.^26^

Previous work has shown that doctors tend to respond rapidly to safety concerns around prescribing, whereas evidence–based guidelines have less impact.^27^^,^^28^ The present findings support this, showing minimal response to the 2014 guidelines, but a rapid reduction in simvastatin 40 mg in 2012, coinciding with a Medicines and Healthcare products Regulatory Agency (MHRA) drug safety alert on simvastatin >20 mg, with corresponding increases in simvastatin 20 mg and atorvastatin. This followed an earlier alert on simvastatin 80 mg due to potential side effects as well as some contraindications for simvastatin.^29^

Cost also contributes to statin choice. Atorvastatin was not recommended widely in the NHS until its patent expired in 2012. Rosuvastatin’s generally low usage over the study period reflects its high cost.

### Implications for research and practice

A recent observational analysis of patients with CVD highlighted the importance of appropriate statin use. Each 10% increase in intensity, for example, 30% to 40%, gave a hazard ratio of 0.90, 95% CI = 0.86 to 0.95, for cardiovascular events.^23^ A combination of prescribing suboptimal statins and imperfect adherence (21% ‘combined measure’) led to an additional 23.7 events per 1000 patient-years above the 48.3 predicted with optimal treatment (high-intensity statins and perfect adherence; 50% ‘combined measure’).^23^

The impact on patient outcomes can be estimated from the widespread use of suboptimal statins identified in the present study. All patients with >10% 10-year risk of CVD are to be offered statins under NICE guidance.^4^ Conservatively assuming an average 15% 10-year risk for the population taking statins, as per the NICE risk calculator, with atorvastatin 20 mg, their 10-year risk would reduce to 9%.^6^ In other words, for every 1000 patients treated with a high-intensity statin, 90 events would be expected over a 10-year period, compared with 150 if untreated, with 60 events prevented. Conservatively assuming a relative risk reduction of 33% for lower-potency statins, only 50 events would be prevented in the same population of 1000 patients at 15% 10-year risk. Therefore, it can be estimated that there will be 10 avoidable cardiovascular events every 10 years for every 1000 patients inappropriately given a lower-potency statin.

In this study, practices exhibiting very rapid changes were identified, demonstrating that greater guideline compliance can be readily implemented. Further work could investigate in detail how this was achieved. The highest high-dose statin prescribing was found in Central London and Bradford, which both have longstanding statin prescribing programmes, with bespoke local guidance, software tools, and incentives.^30^^–^^32^

Having demonstrated that a substantial change in statin prescribing is feasible, the authors suggest that a national strategic approach is required to achieve this: using data to identify outliers, supplying feedback, and targeted educational interventions. Audit and feedback alone are modestly effective at changing clinical practice.^33^ The authors of the present study provide a free, open online data-monitoring tool for high-potency statins — and indeed all medicines — at any individual NHS general practice in England through OpenPrescribing. Two specific areas for further research are identified: first, using data, such as those presented here, to identify practices with the least guideline-compliant statin prescribing and those changing rapidly, and then employing qualitative methods to understand these patterns; second, the evaluation of interventions to improve prescribing, whether low cost, such as simple feedback, or higher cost, such as targeted educational interventions.

The findings of the present study and policy recommendations speak to a more general theme: despite ‘big data’, machine learning, and artificial intelligence commonly discussed as a future panacea in health care, data and analytical techniques, readily available today, are not being used to identify outliers, implement guidance, and improve care. Similarly, if concordance cannot be achieved with the evidence on low-cost, effective, and commonly prescribed statins, then there is a great deal of work to be done in using data and medicines to optimise patient outcomes.
